# Nonuremic Calciphylaxis Precipitated by COVID-19 Infection

**DOI:** 10.7759/cureus.22796

**Published:** 2022-03-03

**Authors:** Hannah Shuman, Mark S Obri, Christina Artz, Raef Fadel, Jonathan Williams

**Affiliations:** 1 Internal Medicine, Wayne State University School of Medicine, Detroit, USA; 2 Internal Medicine, Henry Ford Health System, Detroit, USA; 3 Dermatology, Henry Ford Health System, Detroit, USA

**Keywords:** hypercoagulability, nonuremic calciphylaxis, rheumatology, dermatology, cardiac arrest, calciphylaxis, covid 19

## Abstract

Calciphylaxis is a rare dermatologic condition that is primarily associated with end-stage renal disease (ESRD). Nonuremic calciphylaxis has been reported in patients with autoimmune disorders such as systemic lupus erythematosus and other hypercoagulable states such as anti-phospholipid syndrome. New research throughout the COVID-19 pandemic has shown an increased inflammatory and coagulopathic complication of COVID-19. We present a case of a patient with nonuremic calciphylaxis following treatment for severe COVID-19 and no known cause of hypercoagulability.

A 40-year-old Caucasian female with a history of recent COVID-19 infection requiring hospitalization, hypertension, alcohol abuse, anxiety, and one prior spontaneous miscarriage presented to the hospital with bilateral lower extremity wounds. The wounds were seen to have necrosis and eschar formation, as well as blackened mottled skin, and were extremely painful to the patient. The initial lesions were on the anterior thighs bilaterally and spread laterally and to the lower back. Initial autoimmune workup was non-specific, and biopsy confirmed calciphylaxis.

Calciphylaxis is a known dermatologic disease that has high mortality and morbidity, but it is usually associated with ESRD. Some cases have been reported for autoimmune or hypercoagulable states. The disease presents with non-healing, painful skin ulcers that are at a high risk of infection and have poor healing. The case presented shows biopsy-confirmed calciphylaxis in the absence of known etiologies, and we hypothesize that it is due to COVID-19 or COVID-19 aggravating an underlying but unidentified hypercoagulable condition.

## Introduction

Calciphylaxis is a rare dermatological condition associated with high morbidity and mortality. This condition classically presents with painful, progressive retiform purpura that develops necrotic eschars and is diagnosed via skin biopsy [1,2]. Calciphylaxis is most commonly seen in the setting of end-stage renal disease; however, nonuremic calciphylaxis (NUC) can also occur. Although the exact pathogenesis of NUC remains largely unknown, many disease states are associated with NUC, including autoimmune conditions such as systemic lupus erythematosus and hypercoagulable states such as anti-phospholipid antibody syndrome, antithrombin III deficiency, protein C and S deficiency, and cryofibrinogenemia [1,3-5]. Growing research throughout the COVID-19 pandemic has revealed inflammatory and coagulopathic complications as a result of severe infection [6-8]. We present the case of a patient with NUC in the two months following treatment for severe COVID-19 infection.

## Case presentation

A 40-year-old female with a history of hypertension, alcohol abuse, anxiety, and prior spontaneous miscarriage presented from a skilled nursing facility to an outside hospital with bilateral lower extremity wounds. The wounds initially appeared three weeks prior to presentation as erythematous “sunburn-like” patches that progressed to form blisters, bullae, and necrotic eschars. The initial lesions were located on the anterior thighs bilaterally and subsequently spread laterally and to the lower back. The patient had no family or personal history of autoimmune disease. Home medications included melatonin, clonazepam, fluoxetine, metoprolol, omeprazole, amlodipine, and lisinopril. The patient denied any prior warfarin use.

Of note, the patient had a prolonged hospitalization at an outside hospital about 1.5 months prior to presentation, during which she was treated for acute cardiac arrest secondary to acute hypoxic respiratory failure in the setting of previous COVID-19 infection and superimposed pneumonia. During her admission, the patient had an increasing oxygen requirement due to concern for an acute bacterial pneumonia secondary to COVID-19. The patient was in cardiac arrest requiring chest compressions for 4 minutes before return of circulation was achieved. She was intubated and mechanically ventilated for five days. There was no report of significant renal dysfunction requiring dialysis. The patient was stabilized after 14 days inpatient and subsequently discharged to a skilled nursing facility. The wounds were not present before or during her hospitalization for COVID-19.

**Figure 1:**
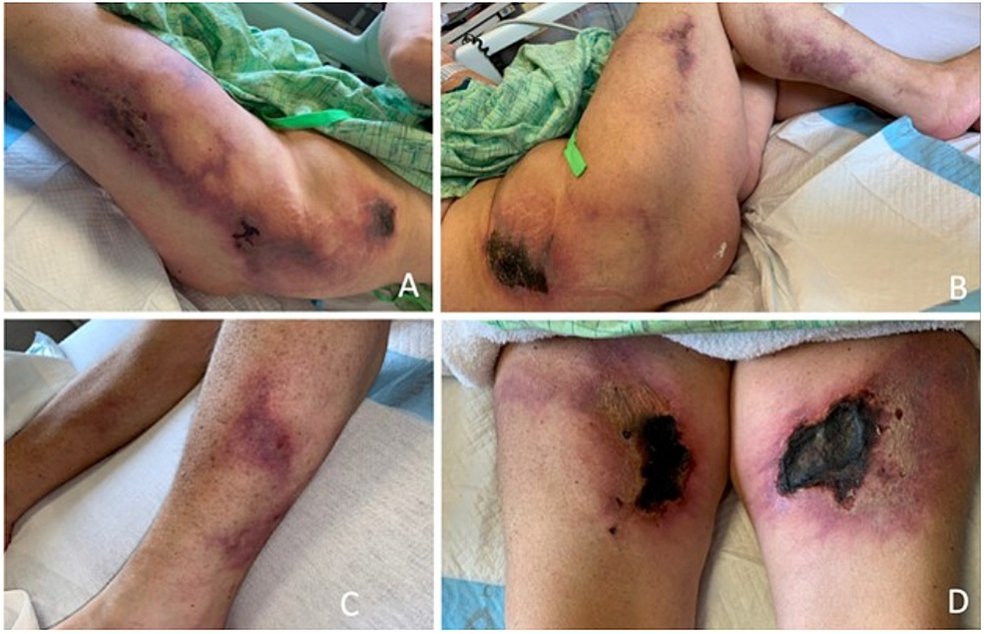
Patient's lower extremities on presentation. (A-C) Early-stage wounds. (D) Progressed wounds with thick eschars within the retiform purpura.

Upon admission to our hospital, the patient was vitally stable. Laboratory evaluation demonstrated hyponatremia, mild leukocytosis, and elevated C-reactive protein and erythrocyte sedimentation rate with otherwise normal kidney function, serum calcium, and parathyroid hormone levels. The patient had a mildly elevated HgbA1C at 5.9% (reference: <5.7 %). Of note, the patient had no significant history of tobacco abuse and no known corticosteroid use except for admission to an outside hospital for her respiratory failure. Physical examination findings demonstrated multiple large indurated retiform purpuras on the bilateral medial and lateral thighs. Medial thighs had large thick eschars centrally located within retiform purpura. Similar thick eschars were present on the bilateral lower lateral hips (Figure 1).

**Figure 2:**
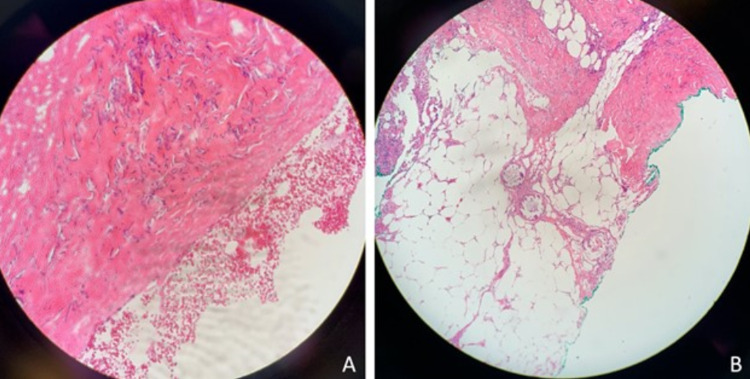
Biopsy results showing calciphylaxis.

A telescoping punch biopsy confirmed the diagnosis of calciphylaxis, with pathology revealing intravascular calcification within subcutaneous adipose tissue and surrounding necrosis (Figure 2).

In light of no underlying renal disease, an extensive autoimmune workup was completed; notable lab values are listed in Table 1. All other autoimmune workup that was performed was negative and is listed in Table 2.

**Table 1:** Autoimmune labs that returned abnormal from complete panel of labs PTT, partial thromboplastin time; PTT-LA, partial thromboplastin time-lupus anticoagulant; DRVVT, diluted Russell viper venom time; ANA: antinuclear antibody; cardiolipin Ab IgM; cardiolipin antibody IgM

Pertinent Lab	Lab Value
Prothrombin time (11.5-14.5 sec)	15.5
PTT (22-36 sec)	43
PTT-LA (reference: <43.5sec)	47.7
DRVVT (reference: 27-45 sec)	54
Lupus anticoagulant (reference: 30.3-43.2sec)	61.2
Hexagonal phospholipid neutralization	Positive
ANA	Positive
ANA pattern	Homogeneous
ANA Titer 1	1:160
Cardiolipin Ab IgM (reference: <12.5MPL)	12.9

**Table 2:** Remaining autoimmune workup that was performed and negative TCT control, thrombin clotting time; RVVNT, Russell viper venom time; ANCA, antineutrophil cytoplasmic antibody; RNP, ribonucleoprotein antibodies; SM, anti-smith; SS A/Ro, Sjögren's-syndrome-related antigen A autoantibodies; SS B/LA, Sjögren's-syndrome-related antigen B autoantibodies; Citr Pep, cyclic citrullinated peptide antibody; SCL-70 (topoisomerase 1); APL, antiphospholipid; GPL, IgG phospholipid units; DAT, direct antiglobulin test

Pertinent Lab	Lab Value
Antithrombin III act (%) (reference: 80-120%)	97
Clot time (sec) (reference: 15-25s)	18.9
TCT control (sec)	22.2
Cryofibrinogen	Absent
B2 glycoproteins IgG, IgM, IgA Ab (SAU) (reference: <2)	<9
RVVNT (reference: <1.2)	1.1
Protein C (reference: 70-140%)	136
Protein S (reference: 55-140%)	82
C-ANCA (titer)	<1:20
P-ANCA (titer)	<1:20
RNP antibody (Elisa units) (reference: <1.0)	<0.2
SM antibody (Elisa units) (reference: <1.0)	<0.2
SS A/Ro antibody (Elisa units) (reference: <1.0)	<0.2
SS B/La antibody (Elisa units) (reference: <1.0)	<0.2
Cyclic Citr Pep, IgG (IU/mL) (reference: <7.0)	<0.4
dsDNA Ab	Negative
Scl-70 Ab (units) (reference: <20)	3
Cardiolipin Ab IgA (APL) (reference: <12)	<9.0
Cardiolipin Ab IgG (GPL) (reference: <12)	<9.0
Direct antiglobulin test (DAT)	Negative

CT of the chest, abdomen, and pelvis was unremarkable for neoplastic etiologies, and lower extremity Dopplers were negative for deep vein thrombosis. The patient was subsequently diagnosed with NUC that was precipitated by COVID-19-induced hypercoagulability. The patient was initiated on sodium thiosulfate (STS) intravenously three times weekly, along with daily wound care, and anticoagulation with apixaban. Due to persistently elevated inflammatory markers and leukocytosis, a wound culture was obtained that subsequently grew many Staphylococcus aureus. The patient was given a 10-day course of cefazolin and metronidazole to cover for suspected wound infection.

**Figure 3:**
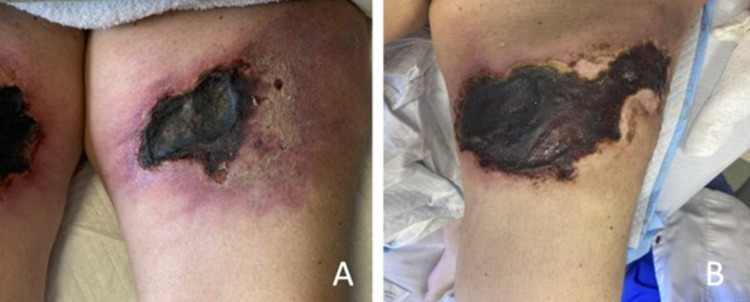
Patient’s left proximal thigh on presentation to our hospital (A) and progression of the left proximal thigh with increasing size of eschar involving prior areas of retiform purpura (B).

Over the hospital course, the damage caused by the calciphylaxis became more declarative, and the initial retiform purpura became large necrotic eschars that continued to spread throughout her lower back (Figure 3). With acceptable pain management and infection control, the patient was discharged to a subacute rehabilitation facility for further long-term management.

## Discussion

A growing body of research suggests COVID-19 infection may lead to a hypercoagulable state that predisposes patients to a myriad of prothrombotic complications [6-8]. Although the detailed pathogenesis is unknown, growing evidence indicates the development of a proinflammatory state that induces hyperviscosity, increasing the patient’s risk of coagulopathy [7,8]. This hypercoagulable state may increase the risk of more common thrombotic complications, including pulmonary embolism, myocardial infarction, and stroke [6]. Cases of a more rare complication, NUC, have also been reported in the setting of COVID-19 [9-11]. Current literature suggests a “two-hit” mechanism wherein a patient may have an asymptomatic or subclinical hypercoagulable condition that, when combined with a prothrombotic state in the setting of severe COVID-19 infection, predisposes patients to rare complications such as calciphylaxis [9,10].

Our patient had no major risk factors for developing NUC. While autoimmune workup revealed elevated antiphospholipid antibodies and our patient had one prior miscarriage, the patient did not meet requirements for a diagnosis of antiphospholipid syndrome. Additionally, her positive autoimmune labs must be placed in the context of a clear pro-inflammatory picture. Additional autoimmune follow-up would be required to determine any chronic disease. The proximity of our patient’s presentation with lower extremity wounds to her previously severe COVID-19 infection would suggest a coagulopathic complication from either a chronic subclinical hypercoagulable state, a complication of COVID-19 infection, or combination of both.

Regardless of the etiology of our patient's NUC, the prognosis was extremely poor and guarded. Consideration of the patient's mental health and priorities is extremely important in managing NUC. The most likely cause of mortality is due to septicemia from the wounds, which necessitates the need for close wound management, likely from a dermatologist/wound care team [12]. In our patient, the wound care team taught the patient management for potential discharge, leading to significantly less anxiety for the patient. Dermatology recommendations were also helpful due to the complexity of the wounds. It was also made clear to the patient that even with the meticulous wound care, it was likely that infection can occur and it was not the fault of the patient.

In addition to the guarded prognosis, extreme pain is a classic symptom of calciphylaxis and is naturally one of the patient's primary concerns. It has been recommended that a multimodal and inter-professional team effort is the most effective way to improve quality of life for the patient [12]. Consulting pain specialists and managing the significant anxiety for our patient was likely beneficial in improving the patient's hospital stay. It was important to set realistic goals and to make the pain as tolerable as possible for our patient while meeting her goals of maintaining as much functionality as she could in this very difficult disease.

## Conclusions

This case report presented a patient with NUC in the setting of recent severe COVID-19 infection. While most cases of NUC are difficult to ascertain the exact etiology, there is a strong association with hypercoagulable states. While the medical community is beginning to acknowledge coagulopathies associated with COVID-19, more research is needed to identify other potential manifestations of COVID-19 coagulopathy, such as calciphylaxis. It is also important to consider a multimodal and inter-professional team for the management of this disease due to the significant burden and poor prognosis. Clear conversations and quality of life discussions should be made a priority with this population of patients.
